# Monitoring M-Protein, Therapeutic Antibodies, and Polyclonal Antibodies in a Multiparametric Mass Spectrometry Assay Provides Insight into Therapy Response Kinetics in Patients with Multiple Myeloma

**DOI:** 10.3390/pharmaceutics17010135

**Published:** 2025-01-19

**Authors:** Charissa Wijnands, Peter G. A. Karel, Jolein Gloerich, Gad Armony, Anastasia Tzasta, Corrie M. de Kat Angelino, Luciano Di Stefano, Vincent Bonifay, Theo M. Luider, Martijn M. VanDuijn, Sandra J. Croockewit, Elizabeth A. de Kort, Daan A. R. Castelijn, Claudia A. M. Stege, Hans J. C. T. Wessels, Alain J. van Gool, Niels W. C. J. van de Donk, Joannes F. M. Jacobs

**Affiliations:** 1 Laboratory Medical Immunology, Department of Laboratory Medicine, Radboud University Medical Center, 6525 GA Nijmegen, The Netherlands; charissa.wijnands@radboudumc.nl (C.W.);; 2 Laboratory of Clinical Chemistry, Deventer Ziekenhuis, 7416 SE Deventer, The Netherlands; 3 Department of Clinical Chemistry, Laboratory Medicine, Unilabs Oost, 7500 KA Enschede, The Netherlands; 4 Translational Metabolic Laboratory, Department of Human Genetics, Radboud University Medical Center, 6525 GA Nijmegen, The Netherlands; jolein.gloerich@radboudumc.nl (J.G.); gad.armony@bruker.com (G.A.); alain.vangool@radboudumc.nl (A.J.v.G.); 5 Sebia, 91090 Lisses, France; ldistefano@sebia.com (L.D.S.); vbonifay@sebia.com (V.B.); 6 Department of Neurology, Erasmus University Medical Center, 3015 GD Rotterdam, The Netherlandsm.m.vanduijn@erasmusmc.nl (M.M.V.); 7 Department of Hematology, Radboud University Medical Center, 6525 GA Nijmegen, The Netherlands; sandra.croockewit@radboudumc.nl (S.J.C.); elizabeth.dekort@radboudumc.nl (E.A.d.K.); 8 Department of Hematology, Amsterdam University Medical Centers, 1081 HV Amsterdam, The Netherlands; d.castelijn@antoniusziekenhuis.nl (D.A.R.C.); n.vandedonk@amsterdamumc.nl (N.W.C.J.v.d.D.); 9 Department of Internal Medicine, St. Antonius Hospital, 3435 CM Nieuwegein, The Netherlands; 10 Department of Hematology, Erasmus Medical Center, 3015 GD Rotterdam, The Netherlands

**Keywords:** multiple myeloma, therapeutic drug monitoring, minimal residual disease, therapy response kinetics, therapeutic antibody, bispecific antibody

## Abstract

**Background/Objectives:** Multiple Myeloma (MM) is a hematologic malignancy caused by clonally expanded plasma cells that produce a monoclonal immunoglobulin (M-protein), a personalized biomarker. Recently, we developed an ultra-sensitive mass spectrometry method to quantify minimal residual disease (MS-MRD) by targeting unique M-protein peptides. Therapeutic antibodies (t-Abs), key in MM treatment, often lead to deep and long-lasting responses. However, t-Abs can significantly decrease the total polyclonal immunoglobulin (Ig) levels which require supplemental IgG infusion. Here, we demonstrate the simultaneous monitoring of M-proteins, t-Abs, and polyclonal Ig-titers using an untargeted mass spectrometry assay, offering a comprehensive view of therapy response. **Methods:** Sera collected between 2013 and 2024 from four patients and cerebrospinal fluid (CSF) from one patient who received various t-Abs were analyzed with MS-MRD. M-protein sequences were obtained with a multi-enzyme de novo protein sequencing approach. Unique peptides for M-proteins and t-Abs were selected based on linearity, sensitivity, and slope coefficient in serial dilutions. Ig constant regions were monitored using isotype-specific peptides. **Results:** The MS-MRD multiplex analysis provided detailed information on drug concentrations and therapy response kinetics. For example, in two patients with refractory disease over five lines of therapy, the MS-MRD analysis showed that the deepest responses were achieved with bispecific t-Ab (teclistamab) treatment. M-protein and t-Ab were also detectable in the CSF of one patient with MS-MRD. **Conclusions:** This proof-of-concept study shows that the multiplex monitoring of the M-protein, any t-Ab combination, and all Ig-isotypes within one mass spectrometry run is feasible and provides unique insight into therapy response kinetics.

## 1. Introduction

Multiple myeloma (MM) is the second most common hematologic malignancy. The disease is characterized by 10% or more clonal plasma cells in the bone marrow (BM) and the production of a monoclonal immunoglobulin (M-protein), accompanied by one or more myeloma-defining events [1]. Novel therapeutics for patients with newly diagnosed or relapsed/refractory disease have substantially improved the survival of MM patients [2,3]. The current standard of care for fit newly diagnosed patients consists of quadruplet induction treatment including a CD38-targeting therapeutic antibody (t-Ab) (e.g., daratumumab), proteasome inhibitor (e.g., bortezomib), immunomodulatory agent (e.g., lenalidomide), and dexamethasone followed by high dose melphalan and autologous stem cell transplantation [3,4,5]. Additionally, first-line treatment with daratumumab, lenalidomide, and dexamethasone has substantially improved the survival of older, non-transplant-eligible patients [6]. Nevertheless, the majority of patients will eventually relapse, highlighting the need for new drugs with novel mechanisms of action.

Continuous advancements in the field of MM therapeutics have led to the development of new agents including T-cell-redirecting bispecific antibodies (bsAbs) which bind simultaneously to CD3 on the T-cell and to an epitope present on MM cells (e.g., BCMA for teclistamab or GPRC5D for talquetamab) [7,8]. Treatment with either teclistamab or talquetamab induced unprecedented deep remissions in heavily pretreated patients with one-fourth of the patients achieving negative minimal residual disease (MRD) status [8,9,10]. Despite these promising results, there are potentially serious adverse events like cytokine release syndrome (CRS) and hypogammaglobulinemia that warrant treatment. A supportive antibody (s-Ab) directed against IL-6 (tocilizumab) is frequently given to manage CRS [11], and supplemental immunoglobulin IgG (IVIg) infusion is recommended to decrease infection burden [9,12]. As treatment with combination agents and t-Abs becomes more frequent, there is a growing need to assess disease status with MRD measurements. The additional monitoring of polyclonal immunoglobulins as well as all the associated t-Abs broadens the scope of therapy monitoring and may ultimately optimize patient care. For instance, information on polyclonal IgG levels can guide the timely initiation of IVIg suppletion to prevent infections. Furthermore, the pharmacokinetic profile of therapeutic antibodies varies from patient to patient, which may lead to significant differences in t-Ab concentrations between patients [13]. In the future, the monitoring levels of circulating t-Abs may enable the prompt adjustment of t-Ab dosage in patients who have subtherapeutic concentrations of the t-Ab. Furthermore, more cost-effective dosing schedules could be established for patients in whom t-Ab concentrations are consistently above the therapeutic threshold.

The International Myeloma Working Group has defined MRD negativity as less than one myeloma cell in 10^5^ nucleated cells in BM [14]. Whilst BM-based MRD assays are capable of sensitive disease monitoring, the procedure itself is highly invasive and patient-unfriendly. The M-protein provides a biomarker that can be detected in the peripheral blood (PB) of MM patients. The current gold standard for M-protein diagnostics consists of serum protein electrophoresis (SPEP) to quantify circulating M-proteins, immunoassays to quantify serum-free light chains, and immunofixation electrophoresis to identify the M-protein isotype [15]. Whilst these PB-based assays provide patient-friendly monitoring tools, sensitivity is insufficient to evaluate MRD status [16,17]. Furthermore, it is a well-known phenomenon that t-Abs can interfere in routine electrophoretic M-protein diagnostics [18,19,20]. To mitigate such interference, electrophoretic hydrashift assays and intact protein mass spectrometry (MS) assays have been developed to correctly differentiate between t-Abs and M-protein [21,22]. Despite these advances, M-protein monitoring remains challenging in patients who reach deep therapy responses when treated with t-Abs and supplemental IVIg, especially when the laboratory specialist is unaware of the exact treatment regimen. To meet these challenges, our group has developed a bottom-up MS-based M-protein monitoring assay (MS-MRD) which is approximately 1000-fold more sensitive than routine M-protein evaluation by SPEP [23,24,25]. Previous MS-MRD studies have demonstrated the ability to sensitively monitor the M-protein in the presence of multiple t-Abs [26].

Recently, we have expanded our MS-MRD assay into a multiplex blood test that allows the ultra-sensitive monitoring of the M-protein, different t-Abs, and all the polyclonal immunoglobulin (Ig) classes in one MS measurement. This study demonstrates a proof-of-concept of the MS-MRD multiplex assay in longitudinally collected sera over a follow-up period of up to eleven years during which the patients have received different t-Abs. The combined dynamic monitoring of MRD activity, therapeutics, and polyclonal Igs using MS-MRD provides unique insights into therapy response kinetics in patients with MM.

## 2. Materials and Methods

### 2.1. Patients and Samples

Four MM patients were selected based on the availability of stored sera and whether they received t-Ab treatment (Table 1). Sera were collected retrospectively from the routine diagnostic laboratory and were stored at −80 °C until use. Sampling intervals varied for each patient and ranged between one and four months. Due to the retrospective nature of this study, not all the samples from the included patients were stored. This resulted in monitoring gaps ranging from one to three years for patients 2 and 3. For patient 4, both sera and cerebral spinal fluid (CSF) samples were collected. The CSF samples were collected through a lumbar puncture between the 3rd and 4th lumbar vertebrae and stored at −80 °C until use. Written informed consent was provided by the patients and clinical data were de-identified in accordance with the Declaration of Helsinki. Routine M-protein monitoring was performed with SPEP on all the samples of patients 2 and 4. Patients 1 and 3 were monitored using the Freelite free light chain assay (The Binding Site, Birmingham, UK).

Ten control sera were collected from chronic kidney disease (CKD) patients and pooled together. This serum pool was used as a matrix for serial dilution experiments. CKD patients have elevated levels of polyclonal Igs, and using these pooled sera as the matrix for peptide selection improves the exclusion of non-unique M-protein peptides.

### 2.2. Sample Preparation and Digestion

Sera and CSF samples were diluted 250-fold in HPLC grade water with 7.5% acetonitrile (ACN) (Biosolve, Valkenswaard, The Netherlands) and 35 mM ammonium bicarbonate (Sigma-Aldrich, St. Louis, MO, USA). To 10 µL of the diluted serum, 1 ng/µL SILu™MAB K1 (SILuMAB) (Sigma-Aldrich, St. Louis, MO, USA) was added. Proteins were denatured by diluting the samples 1:1 in 0.2 *w*/*v*% RapiGest SF Surfactant (Waters, Milford, MA, USA), and 0.7 *w*/*v*% 0.1 M dithiothreitol (Sigma-Aldrich, St. Louis, MO, USA) was added to reduce cystine disulfide bridges. The samples were incubated for 30 min at 60 °C in an Eppendorf™ ThermoMixer while mixing at 1× *g*. To alkylate cysteine reduced side chains, 0.04 *w*/*v*% 0.3M iodoacetamide (Sigma-Aldrich, St. Louis, MO, USA) was added. The samples were incubated in the dark for 20 min at room temperature. Sequencing grade trypsin (Promega, Madison, WI, USA) was added in a concentration of 70 ng/µg protein and incubated at 37 °C overnight. The digestion was stopped using 0.7 *v*/*v*% trifluoroacetic acid (Biosolve, Valkenswaard, The Netherlands).

### 2.3. LC-MS/MS Acquisition and Data Processing

Peptides were separated on an Evosep one LC system using C18 Evotips (Evosep, Odense, Denmark). Per measurement, the equivalent of 100 ng total protein of digested serum was loaded per the manufacturer’s instructions. Peptide separation was performed on an 8 cm C18 column (Evosep Performance, ReproSil-Pur C18, 150 µm I.D., 1.5 µm particle size) and eluted with a linear gradient of buffer A (HPLC grade water with 0.1% formic acid) and buffer B (ACN with 0.1% formic acid) according to the Evosep 60SPD method. The eluted peptides were analyzed on a timsTOF Pro 2 (Bruker Daltonics, Bremen, Germany) operated in the Parallel Accumulation-SErial Fragmentation (PASEF) mode. Prior to recording the Data-Independent Acquisition (DIA)-PASEF measurements, Data-Dependent Acquisition (DDA) measurements were performed and processed using a Parallel search engine in real time (PaSER) (Bruker Daltonics, Bremen, Germany). The data were searched against a database of the secreted human proteome (UniProt) supplemented with patient-specific M-protein and SILuMAB sequences using ProLuCID [27]. Searches were performed within the precursor mass range of 400–6000 *m*/*z*. Precursor and fragment mass tolerances were set to 20 and 30 ppm, respectively. The false positive rate was set to 0.01 with a maximum of two missed cleavages. From the DDA searches, spectral libraries were constructed in PaSER and coupled to the DIA method to facilitate real-time data processing. In the DIA mode, MS/MS scans were recorded within a *m*/*z* range of 400–1000 and an ion mobility range of 0.6–1.43 1/K0. Precursors were selected for fragmentation within windows of 5 *m*/*z*, and 0.3 1/K0 with increments of 0.02 1/K0 for each window. The DIA measurements were processed using PaSER and analyzed with Trapped Ion Mobility Spectrometry Data-Independent Acquisition–Neural Network (TIMS DIA-NN) [28] in real time.

### 2.4. M-Protein Sequencing and Clonotypic Peptide Selection

M-protein sequences were identified by the M-inSight (Sebia, Lisses, France) multi-enzyme de novo sequencing approach [29,30]. To select clonotypic peptides, serial dilutions were prepared from patient sera diluted in pooled CKD control serum as demonstrated before [24]. In short, clonotypic peptides are patient-specific and should dilute in a linear fashion. Serial dilutions were prepared from the tryptic digests of MM patient sera in pooled control serum in six subsequent ten-fold or five-fold dilutions depending on the M-protein concentration. To select peptides for the t-Abs and s-Abs, all the antibodies used in the treatment of the four included patients (daratumumab, isatuximab, talquetamab, teclistamab, and tocilizumab) were spiked in the pooled CKD control sera at 1 g/L and digested with trypsin. The tryptic digest was serially diluted in a tryptic digest of the pooled CKD control sera without t-Abs and s-Abs in six subsequent five-fold dilutions. M-protein, t-Ab, and s-Ab peptides were selected based on the lowest detectable concentration (≤0.02 g/L), linearity (R^2^ ≥ 0.7), and slope coefficient (−0.7 to −1.3).

### 2.5. MS-MRD Assay Sensitivity

Assay sensitivity was determined for each analyte separately by the assessment of the limit of detection (LoD) and lower limit of quantification (LLoQ). The LoD was defined as (3.3 × SD of control serum)/slope, and LLoQ was defined as 3 × LOD as described by the ICH Q2(R2) guideline on the validation of analytical procedures (version effective from 14 June 2024, http://www.ich.org, accessed on 15 December 2024).

### 2.6. Peptide Selection for the Monitoring of Polyclonal Immunoglobulins

Peptides derived from IgG1, IgG2, IgG3, IgG4, IgA1, IgA2, IgM, IgD, and IgE constant regions were compared to a database of the human-secreted proteome (UniProt). Unique peptides were analyzed in the pooled CKD control serum and the top three peptides based on the highest peak area per Ig (sub)class were selected for the monitoring of polyclonal Igs. Additionally, common IgG and IgA peptides were selected to monitor the total IgG and IgA.

### 2.7. M-Protein, t-Ab, and Polyclonal Immunoglobulin Quantification in Follow-Up Sera and CSF

The method used for M-protein quantification in the follow-up samples was described previously [25]. In short, clonotypic peptide peak areas were normalized on averaged SILuMAB peak areas. Relative M-protein concentrations were then converted to absolute concentrations by calibrating on a known M-protein concentration measured by SPEP. The following formula was applied:

(1) M_f=(C_f/S_f)/(C_b/S_b)×M_b

in which M = M-protein (g/L); C = raw peak area of clonotypic peptide; S = average peak area of SILuMAB peptides; f = follow-up time point; and b = baseline time point. Consecutive follow-up data points were connected using spline interpolation.

T-Ab and s-Ab concentrations in the patient sera and CSF were quantified in a similar fashion where the required known reference concentration was generated by spiking t-Abs and s-Abs in a patient sample prior to t-Ab treatment at a concentration of 1 g/L. Reference values for the total IgG, IgA, and IgM were obtained by immunoturbidimetric measurements using a Cobas8000 (Roche, Basel, Switzerland), and reference values for the IgG subclasses were obtained by nephelometric measurements using an Atellica Solution (Siemens, Munich, Germany). These measurements were performed per the manufacturer’s instructions as a part of the routine diagnostic work-up.

## 3. Results

### 3.1. Patient Cohort and Therapy Response

Four patients were included in this study: patients 1 and 3 had FLC-kappa M-proteins, whilst patients 2 and 4 had IgA-kappa and IgG-kappa M-proteins, respectively (Table 1). Therapy response was assessed according to the response criteria defined by the International Myeloma Working Group [14]. All the patients received multiple t-Abs as part of different treatment lines for their MM; all the patients received CD38-targeting antibodies and teclistamab, and patient 4 also received talquetamab. Prior to the t-Ab treatment, all the patients had received several lines of therapy, including one or two autologous stem cell transplants (ASCTs). Response assessment showed variable reactions to the different therapeutic regimes among all the patients. However, a general trend was observed, suggesting that later treatment lines were less effective than earlier treatment lines. An exception to this pattern was observed with bsAbs in two patients. Notably, patients 1 and 2 achieved a stringent complete response (sCR) following the initiation of the bsAb treatment (most recent treatment line; 9th and 6th line, respectively).

### 3.2. Clonotypic Peptide Selection of M-Proteins, t-Abs, and s-Abs

The identification of the M-protein sequence is crucial for M-protein monitoring using MS-MRD. Using a multi-enzyme de novo sequencing approach (M-inSight, Sebia, Lisses, France), all the M-protein sequences were successfully identified. The protein sequences of daratumumab, talquetamab, teclistamab, and tocilizumab were obtained from Drugbank Online (https://go.drugbank.com/, accessed on 1 November 2024). The protein sequence of isatuximab was obtained from the KEGG drug database (https://www.genome.jp/kegg/drug/, accessed on 1 November 2024). One or more clonotypic peptides were successfully identified from the variable region of each patient, t-Ab, and s-Ab (Table S1).

Using the selected clonotypic peptides, the LoD and LLoQ of all the M-proteins, t-Abs, and s-Abs were determined (Table 2). The LoD of the M-proteins ranged from 0.0003 g/L to 0.0005 g/L, and from 0.0001 g/L and 0.0006 g/L for the t-Abs and s-Abs. The LLoQ of the M-proteins ranged from 0.0009 g/L to 0.0014 g/L, and from 0.0001 g/L and 0.0017 g/L for the t-Abs and s-Abs. The LoD and LLoQ of M-protein, t-Abs, and s-Abs in CSF could not be determined due to the lack of CSF control samples.

### 3.3. M-Protein Monitoring

**Figure 1:**
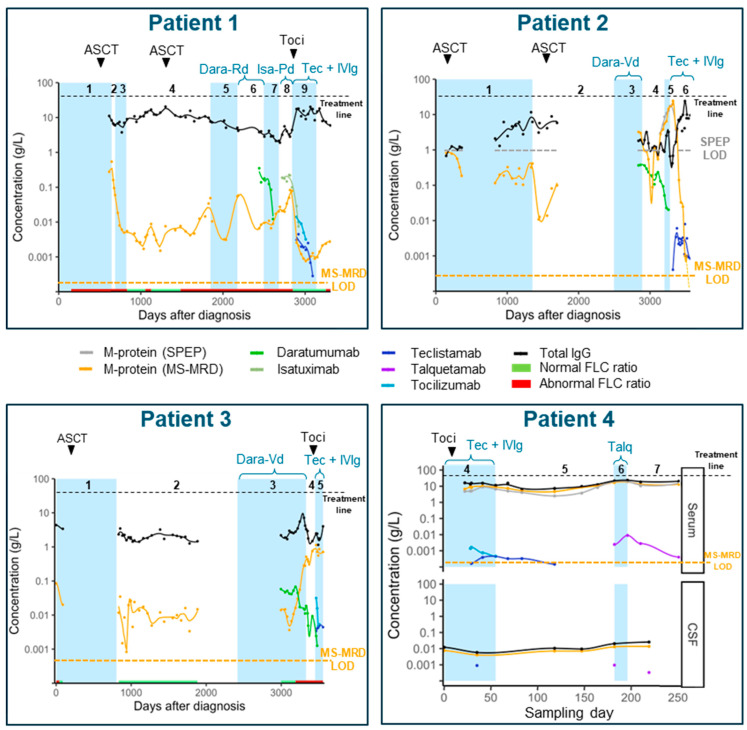
Dynamic M-protein, t-Ab, and total IgG levels in the sera of four patients and CSF samples of one patient. The different therapy lines are indicated by blue and white colored areas that are numbered above each graph. The details of each line of therapy are shown in Table 1. The LoD for each M-protein is indicated by the orange dashed line. For patients 2 and 4, routine M-protein monitoring was performed using SPEP. For patients 1 and 3, FLC assay was used to routinely monitor the M-protein. FLC status is indicated on the x-axis of patients 1 and 3. For patient 4, follow-up sera and CSF samples were available from the 4th line of therapy. ASCT: autologous stem cell transplantation; Dara: daratumumab; Isa: isatuximab; IVIg: intravenous immunoglobulins; Talq: talquetamab; Tec: teclistamab; Toci: tocilizumab; CSF: cerebrospinal fluid; LOD: limit of detection.

Follow-up samples from patients 1, 2, 3, and 4 included sera collected in 2016, 2014, 2013, and 2023, respectively, until 2024. These samples cover follow-up periods of eight, ten, eleven, and two years and originated from patients who have had numerous lines of therapy (nine, six, five, and seven lines, respectively). In all the patients, MS-MRD and the selected clonotypic peptides allowed for the dynamic monitoring of M-protein levels, depicted by the orange lines in Figure 1. All the serum samples of patients 2 and 4 were monitored with SPEP (gray lines in Figure 1). In all the serum samples from patient 4, the M-protein could be quantified using SPEP. However, in patient 2, only samples between day 3000 and 3218 tested positive with SPEP. In the samples with measurable M-protein levels by SPEP, the M-protein concentrations corresponded to M-protein concentrations measured with MS-MRD (r = 0.901). Patients 1 and 3 had a FLC-kappa-producing MM Both patients were monitored by using a nephelometric FLC assay instead of SPEP measurements. Using MS-MRD, it was possible to quantify the M-protein even after the SPEP and FLC levels normalized in patients 1, 2, and 3. Clonotypic peptides were detected in all the sera except for the seven most recent samples of patient 2, which could be attributed to a deep state of remission. The observed M-protein dynamics generally aligned with response to therapy across all the patients. An M-protein decrease was generally observed after the start of each therapy line, followed by an increase when therapy was stopped due to disease progression. Furthermore, the MS-MRD analysis offered more sensitive information on disease activity compared to routine M-protein evaluation, providing deeper insights into therapy response. Notably, both patients 1 and 2 reached the deepest response with teclistamab out of all the earlier therapy lines which included two courses of ASCT and anti-CD38 antibody-based regimens. These findings corresponded with the clinical assessment in which the most recent line of therapy was the most effective for both patients 1 and 2 (sCR) (Table 1). However, due to the development of myelodysplastic syndrome, teclistamab treatment was discontinued in patient 1, and five months after discontinuation, an increase in serum-free light chain levels was observed, indicating relapse. In contrast to the response of patients 1 and 2, the dynamic M-protein monitoring of patients 3 and 4 revealed the complete absence of a response to teclistamab. Table S2 shows an overview of all the MS-MRD values in relation to other diagnostics performed in each sample. 

Due to the development of meningeal involvement (myelomatous meninigitis), cerebrospinal fluid (CSF) samples were collected at six time points in patient 4, of which the baseline sample was taken prior to starting teclistamab. In both sera and CSF, the MS-MRD analysis revealed limited dynamic changes in M-protein levels during treatment with teclistamab + pomalidomide + cyclophosphamide and talquetamab (refractory disease). The serum M-protein levels of patient 4 remained also stable with these therapeutic regimens. M-protein levels in serum were consistently ~1000-fold higher compared to CSF.

### 3.4. M-Protein Kinetics

Frequent MS-MRD measurements enable the evaluation of M-protein dynamics. As an example, the dynamic reduction in M-protein levels during each line of therapy was determined for patient 1 (Table 3). All the therapies were ranked according to the M-protein concentration and slope steepness. These results corresponded to the therapy responses shown in Table 1 in which lines 4 and 9 demonstrated the highest effectiveness, whilst lines 7 and 8 showed the least effectiveness. These data suggest that the steepness of the descending slope in combination with the depth of the response is most indicative of successful therapy responses. However, evaluating M-protein descending slopes is more complex than assessing response depth, as slopes depend on the M-protein isotype. In patients with FLC-type M-proteins, such as patient 1, M-protein clearance occurs rapidly. Consequently, it is expected that slope steepness has the most predictive value in FLC patients. However, this predictive value of slope steepness should be studied further in larger cohorts.

### 3.5. Monitoring Therapeutic and Supportive Antibodies

All the t-Abs and s-Abs administered to the patients were detected in both the serum and CSF follow-up samples. t-Ab-specific mass spectrometry signals were exclusively detected after the specific t-Ab administration. These results confirm the uniqueness of the selected peptides and resulted in the high sensitivity and the 100% specificity that was observed. In all the patients, a 100-fold difference in concentration was observed between anti-CD38 t-Abs (daratumumab and isatuximab) and bsAbs (talquetamab and teclistamab) which corresponds to the difference in dosing. Tocilizumab was detected in the patients who received this anti-IL-6 receptor-blocking antibody for the treatment of cytokine-release syndrome (Figure 1).

The average ratio of teclistamab and talquetamab concentrations in CSF and PB was 1:10. The detection of bsAbs in CSF was unexpected given the relatively low treatment dose. However, the protein composition of CSF is different and less complex compared to serum, which may explain the detection of bsAb-specific signals in CSF. Since bsAb concentrations are close to the MS-MRD limit of quantification, the discrepancy in the PB:CSF ratio between M-protein (1:1,000) and bsAbs (1:10) could be partially attributed to assay linearity challenges at very low analyte concentrations.

### 3.6. Half-Life of Therapeutic Antibodies and Supportive Antibodies

All the t-Abs, s-Abs, and bsAbs analyzed in this cohort are humanized IgG immunoglobulins, of which the nominal half-life (t1/2) is approximately 21 days [31]. In patient 1, the t1/2 of all the t-Abs and s-Abs was determined based on the observed concentrations after the therapy administration was stopped (Table S3). The results show a t1/2 twice as high for anti-CD38 antibodies (43 days for daratumumab and 35 days for isatuximab) compared to the nominal IgG t1/2. For patients 2 and 3, the observed daratumumab t1/2 was even longer (91 and 60 days, respectively).

The clearance of any Ig, including t-Abs, is influenced by interaction with the Ig-binding target. For this reason, it could be argued that the clearance of t-Abs depends on the availability of its target which is mainly influenced by the presence of myeloma cells. Patients 1 and 3 had a FLC-Kappa M-protein of which the t1/2 is extremely short (2–4 h). For this reason, changes in FLC-type M-proteins resemble the tumor dynamics most accurately. Statistical power was insufficient for an accurate analysis. However, a moderate correlation is suggested between the M-protein concentration at the treatment stop and t-Ab t1/2 for the two FLC patients in this cohort (r = 0.502). On the other hand, IgG-type M-proteins may result in faster t-Ab clearance due to the saturation of the neonatal Fc receptor, which aids in IgG reabsorption. However, there was insufficient data available for statistical analysis.

### 3.7. Monitoring Polyclonal Immunoglobulins

To monitor the total Ig levels per (sub)class, unique peptides were selected for the constant regions of all the Ig (sub)classes. Screening of the top three peptides based on peak area resulted in the selection of peptides for all the (sub)classes which are shown in Table S1. With the exception of IgE, all the selected Ig peptides could be monitored in all the patients. IgE could not be detected in the patient sera or CSF, most likely because of its low serum concentration.

The depletion of the total Ig levels is a recognized side effect that is associated with t-Ab treatment and, in particular, with bsAb therapies [31]. A decrease in Ig levels of all the classes which coincided with the start of t-Ab treatment was observed in all the patients. Figure S1 shows these findings in more detail in patient 1. Following Dara-Rd (daratumumab, lenalidomide, dexamethasone) treatment, the total Ig levels declined. This decrease persisted with the subsequent administration of isa-Pd (isatuximab, pomalidomide, and dexamethasone), and the total Ig levels even further decreased with teclistamab monotherapy. This trend of immunoparesis was discontinued by a sharp increase in IgG specifically, which concurred with IVIg IgG supplemental infusion. These results reflect the endogenous humoral antibody status of an individual patient and may guide a clinician on decisions regarding IgG supplementation. Finally, it is important to note that the MS-MRD multiplex assay cannot differentiate between endogenous polyclonal IgG and the supplemental IgG from the IVIg product.

## 4. Discussion

Therapy response assessment is crucial to monitor therapy effectiveness in patients with MM. In the routine clinical setting, response depth is mainly based on the M-protein levels determined by electrophoresis and free light chain ratios determined by nephelometric analysis in serum and/or urine [14]. Deeper responses can be monitored by MRD measurements in BM aspirates. Previously, serum-based MS-MRD was shown to provide approximately the same sensitivity as NGS-MRD on BM [32]. The ability to frequently monitor patients using PB provides sensitive information on the dynamics of the M-protein. In combination with the depth of response, the slope of the M-protein decline could be used to gain insights into therapy response kinetics. For instance, a fast and deep response following the initiation of teclistamab treatment was observed in two of the four patients monitored in this study. For these two patients, the deepest response since diagnosis was achieved with teclistamab treatment even after five and eight lines of prior treatment. Patient 1 developed disease progression five months after the teclistamab treatment was discontinued due to the development of myelodysplastic syndrome. Patient 2 remained on the teclistamab treatment and continued to respond up to the point that even the MS-MRD results turned negative during this follow-up period. In contrast, patients 3 and 4 showed progressive disease despite the teclistamab treatment. Whilst reliable comparison to other studies is impossible due to the small cohort size, the response rate found in our study does correspond to the findings in clinical trials [9,10]. To determine the clinical value of M-protein slopes, validation in larger cohorts is needed.

In this study, we demonstrated the ability to monitor the M-proteins during long follow-up periods of up to ten years which covered multiple relapses and remissions. This demonstrated the stability of the M-proteins as a biomarker for MM disease activity. On a genetic level, Langerhorst et al. demonstrated the stability of the M-proteins as a biomarker throughout disease progression [33]. We were able to reproduce these findings on a protein level which further underscores the stability of clonotypic M-protein peptides for the purpose of disease monitoring.

M-proteins and t-Abs were quantified in both PB and CSF. Previous studies have shown the ability to monitor multiple t-Abs including daratumumab in serum [26,34]. Here, we demonstrate the monitoring of T-cell-redirecting bsAbs which are administered in concentrations that are approximately 100-fold lower compared to the anti-CD38 antibody dosing. The ability to monitor both M-proteins and t-Abs in different liquid matrices could be useful in monitoring patients with CNS involvement or in the assessment of the degree of blood–brain barrier penetrance of biological substances.

For both daratumumab and isatuximab, the observed clearance was longer compared to the other t-Abs analyzed in this study and the nominal IgG t1/2 of ~21 days. The described clearance of isatuximab is 57 days in non-IgG MM [35], which roughly corresponds to the t1/2 of 43 days observed in patient 1. A possible explanation for this slower clearance could be CD38 expression by red blood cells which causes an interaction between daratumumab/isatuximab and red blood cells [36]. Additionally, variation in the clearance of daratumumab was observed between patients. This inter-patient variability and non-linear clearance have been described previously and were partly attributed to variations in target burden [37]. The median teclistamab clearance observed in the MajecTEC-1 trial was 15 days [38], whilst we observed a slower clearance (24, 31, and 38 days). However, reliable comparison is complicated since the clearance was not reported for the IgG and non-IgG MM groups.

Selecting the right peptide targets to monitor the M-proteins, t-Abs, and s-Abs that are both unique and can be monitored sensitively is a crucial step in the MS-MRD workflow. BsAbs consist of two different arms which provide more potential clonotypic peptides. However, this could also impact assay sensitivity since the abundance of each peptide is twice as low. For talquetamab, we could not identify peptide targets from the anti-GPRC5 arm, which means that there are no truly unique peptide targets for talquetamab generated by tryptic digestion. In this study, talquetamab and teclistamab were not administered simultaneously, and peptides shared with teclistamab from the anti-CD3 arm could be used to monitor talquetamab. However, clinical trials are currently investigating the effectiveness of talquetamab and teclistamab combination therapy which could hamper talquetamab monitoring by MS-MRD [39]. One potential solution for the simultaneous monitoring of talquetamab and teclistamab is to explore the use of alternative digestion enzymes. These enzymatic cleavages would result in different peptides from the anti-GPCR5 arm and could potentially produce unique peptides.

In conclusion, we demonstrated the ability of MS-MRD to monitor the M-proteins, t-Abs, s-Abs, and polyclonal Ig levels in one untargeted mass spectrometry assay in the blood of patients as well as their CSF. The modular design of MS-MRD allows for the incorporation of novel therapeutics without the need to modify or develop a novel assay. This multiplex MS-MRD assay provides unique insights into therapy response kinetics in patients with MM.

## Figures and Tables

**Table 1:** Response to therapy for each line of therapy described per patient. T-Abs are indicated in bold text. V: bortezomib; C: cyclophosphamide; D or d: dexamethasone; HDM: high-dose melphalan; ASCT: autologous stem cell transplantation; REP: lenalidomide + cyclophosphamide + prednisone; R: lenalidomide; Ixa: Ixazomib; Dara: daratumumab; Iber: Iberdomide; Isa: isatuximab; P: pomalidomide; T: thalidomide; K: Carfilzomib; mono: monotherapy; VGPR: very good partial response; sCR: stringent complete response; PR: partial response; SD: stable disease; PD: progressive disease.

Patient	M-Protein	Therapy	Therapy Line	Therapy Response
1	FLC-Kappa	VCD + HDM + ASCT	1	VGPR
		Vd	2	VGPR
		REP	3	PR
		HDM + ASCT, R maintenance	4	sCR
		Ixa-D	5	VGPR
		Dara-Rd	6	VGPR
		C-Iber-D	7	SD
		Isa-Pd	8	SD
		Teclistamab mono	9	sCR
2	IgA-Kappa	VCD + HDM + ASCT +VRd (consolidation), R maintenance	1	sCR
		VTD + HDM + ASCT	2	sCR
		Dara-Vd, dara mono	3	PR
		Pom-Cd, pom mono	4	VGPR
		Pom-C	5	PD
		Teclistamab mono	6	sCR
3	FLC-Kappa	VCD + HDM + ASCT, R maintenance	1	sCR
		KPd, Pd maintenance	2	sCR
		Dara-Vd, dara mono, dara-R	3	sCR
		C-Iber-D	4	SD
		Teclistamab mono	5	SD
4	IgG-Kappa	VCD, HDM + ASCT, R maintenance	1	VGPR
		Isa-Pd	2	PD
		KCd	3	VGPR
		Teclistamab mono	4	PD
		Pom-C	5	PR
		Talquetamab mono	6	PD
		Pom-C	7	PD

**Table 2:** Serum LoD, LLoQ, R^2^, and slope for each analyte monitored in this study. *LoD: Limit of detection; LLoQ: Lower limit of quantification*.

Analyte	LoD (g/L)	LLoQ (g/L)	R^2^	Slope
M-protein patient 1	0.0003	0.0009	0.992	−0.884
M-protein patient 2	0.0004	0.0011	0.995	−0.780
M-protein patient 3	0.0005	0.0014	0.969	−0.801
M-protein patient 4	0.0003	0.0010	0.985	−0.790
Daratumumab	0.0006	0.0017	1.000	−0.913
Isatuximab	0.0004	0.0013	0.997	−0.877
Talquetamab	0.0006	0.0010	0.972	−0.885
Teclistamab	0.0002	0.0006	0.972	−0.885
Tocilizumab	0.0001	0.0003	0.998	−0.934

**Table 3:** M-protein reduction and depth of response calculated for each line of therapy in patient 1. Slope is expressed in log10(M-prot(%))/time(year). M-protein levels were determined by MS-MRD.

Treatment Line	Slope	Lowest M-Protein (g/L)
1	no data	no data
2	−8.2	0.0599
3	−2.5	0.0050
4	−2.2	0.0016
5	−2.1	0.0032
6	−1.3	0.0066
7	−1.9	0.0067
8	−1.5	0.0209
9	−4.9	0.0008

## Data Availability

The data presented in this study are available upon request from the corresponding author due to privacy reasons.

## Supplementary Materials

The following supporting information can be downloaded at https://www.mdpi.com/article/10.3390/pharmaceutics17010135/s1, Figure S1: Longitudinal analysis of all immunoglobulin isotypes in patient 1; Table S1: Selected peptides to monitor M-proteins, t-Abs, and all classes and subclasses of total immunoglobulin levels in all patients; Table S2: Overview of all collected samples and diagnostic tests performed on each sample; Table S3: Half-life calculated for each t-Ab administered to each patient.
