# Supplementary material for: Monitoring M-Protein, Therapeutic Antibodies, and Polyclonal Antibodies in a Multiparametric Mass Spectrometry Assay Provides Insight into Therapy Response Kinetics in Patients with Multiple Myeloma
Source: Pharmaceutics. 2025 Jan 19;17(1):135. doi: 10.3390/pharmaceutics17010135 (PMC11769374; doi:10.3390/pharmaceutics17010135)
Supplement: Supplementary file 1 [file pharmaceutics-17-00135-s001.zip › pharmaceutics-3327013-supplementary.pdf]

Figure S1. Longitudinal analysis of all immunoglobulin isotypes in patient 1.
Dara: Daratumumab; Isa: Isatuximab; Tec: Teclistamab; IVIg: Intravenous immunoglobulins

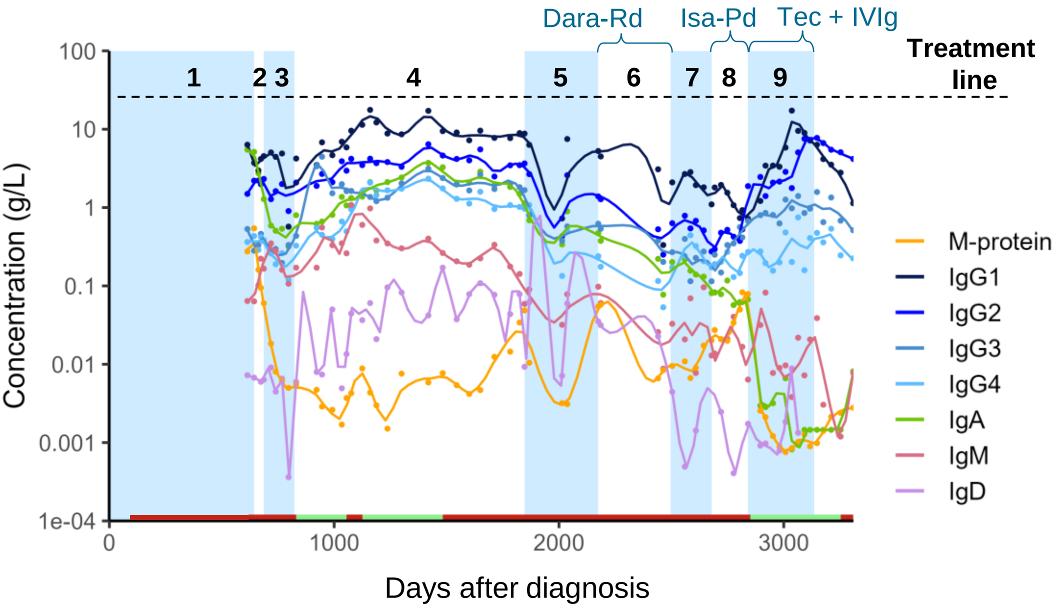

Table S1. Selected peptides to monitor M-proteins, t-Abs, and all classes and subclasses of total immunoglobulin levels in all patients.

LC: light chain; HC: Heavy chain; t-Ab: Therapeutic antibody; s-Ab: supportive antibody; Ig: Immunoglobulin

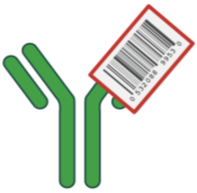

M-protein, t-Ab, and s-Ab: target in Fab

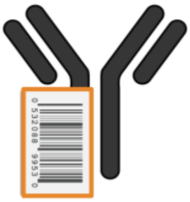

Total Ig per isotype: target in Fc

Analyte	Selected peptides	m/z	z
Patient 1 LC	ASTLEAGVPSR	543.7871	2
Patient 2 HC	LSC^{CAM}VASGFTFSSYVMHWVR	744.684	3
	NTVFVQMNSLRPEDTAVYYC^{CAM}AR	878.0813	3
Patient 2 LC	LLISGVSSR	465.7786	2
Patient 3 LC	LLIYDASNLETGVPSR	873.9612	2
Patient 4 HC	SSYFWGWIR	600.7895	2
Patient 4 LC	DIVMTQSPATLSVSPGER	943.974	2
	ASQSVGGSLAWYQQKPGQAPR	738.7069	3
Daratumumab HC	GLEWVSAISGSGGGTYYADSVK	734.6835	3
Isatuximab HC	QRPGQGLEWIGTIYPGDGDTGYAQK	902.437	3
	TVYMHLSSLASEDSAVYYC^{CAM}AR	807.7002	3
Isatuximab LC	ASQDVSTVVAWYQQKPGQSPR	1166.084	2
	DIVMTQSHLSMSTSLGDPVSITC^{CAM}K	869.0821	3
Talquetamab anti-CD3 HC	HGNFGNSYVSWFAYWGQGTLVTVSSASTK	1050.497	3
	LSC^{CAM}AASGFTFNTYAMNWVR	1098	2
	YNNYATYYAASVK	509.2371	3
Talquetamab anti-CD3 LC	SSTGAVTTSNYANWVQQKPGQAPR	849.418	3
Teclistamab anti-CD3 HC	HGNFGNSYVSWFAYWGQGTLVTVSSASTK	1050.497	3
	LSC^{CAM}AASGFTFNTYAMNWVR	1098	2
	YNNYATYYAASVK	509.2371	3
Teclistamab anti-CD3 LC	SSTGAVTTSNYANWVQQKPGQAPR	849.418	3
Teclistamab anti-BCMA LC	SVHWYQQPPGQAPVVVVYDDSDRPSGIPER	844.6625	4
	VEAGDEAVYYC^{CAM}QVWDSSSDHVVFGGGTK	1020.782	3
Tocilizumab HC	LLIYYTSR	514.2888	2
Tocilizumab LC	TTAMDYWGQGSLVTVSSASTK	1095.019	2
IgG1	EPQVYTLPPSR	428.8913	3
	THTC^{CAM}PPC^{CAM}PAPELLGGPSVFLFPPKPK	711.1144	4
IgG2	C^{CAM}C^{CAM}VEC^{CAM}PPC^{CAM}PAPPVAGPSVFLFPPKPK	727.1004	4
	VVSVLTVVHQDWLNGK	896.9954	2
IgG3	SC^{CAM}DTPPPC^{CAM}PR	593.249	2
	TPLGDTTHTC^{CAM}PR	677.8186	2
IgG4	EPQVYTLPPSQEEMTK	938.4499	2
	GLPSSIEK	415.2309	2
	WQEGNVFSC^{CAM}SVMHEALHNHYTQK	934.0837	3
Total IgG	DTLMISR	417.7171	2
	NQVSLTC^{CAM}LVK	580.8148	2
IgA1	DASGVTFTWTPSSGK	770.3639	2
	NFPPSQDASGDLYTTSSQLTLPATQC^{CAM}LAGK	1056.172	3
	TFTC^{CAM}TAAYPESK	687.8099	2
IgA2	DASGATFTWTPSSGK	756.3483	2
	NFPPSQDASGDLYTTSSQLTLPATQC^{CAM}PDGK	1065.491	3
Total IgA	GDTFSC^{CAM}MVGHEALPLAFTQK	552.2604	4
	QEPSQGTTTFAVTSILR	917.9749	2
	VAAEDWK	409.2022	2
IgD	APDVFPIISGC^{CAM}R	665.8388	2
	DSYYMTSSQLSTPLQQWR	1095.506	2
	VPAPPSPQPATYTC^{CAM}VVSHEDSR	798.3782	3
IgM	DVMQGTDEHVVC^{CAM}K	758.8362	2
	ESDWLGQSMFTC^{CAM}R	808.3416	2
	YAATSQVLLPSK	638.855	2
IgE	VAHTPSSTDWVDNK	778.367	2
	TYTC^{CAM}QVTYQGHTFEDSTK	1082.972	2
	DWIEGETYQC^{CAM}R	728.3081	2

Table S2: Overview of all collected samples and diagnostic tests performed on each sample.*(1/6)*

Patient ID	Sample	Collection date	M-prot (SPEP) (g/L)	FLC Kappa (g/L)	FLC Lambda (g/L)	FLC K/L ratio	IgG (g/L)	IgA (g/L)	M-prot (MS-MRD) (g/L)	Storage time (months)
Patient 1	Serum	0	Traces							Not stored
Patient 1	Serum	78		0.0292	0.0033	8.85				Not stored
Patient 1	Serum	180		0.0471	0.0068	6.93				Not stored
Patient 1	Serum	610		3.1800	0.0036	883			0.2756	213
Patient 1	Serum	640		11.7000	0.0025	4680			0.5427	210
Patient 1	Serum	659		0.5790	0.0026	223			0.1605	209
Patient 1	Serum	670		0.9230	0.0039	237			0.0944	208
Patient 1	Serum	683		0.0599	0.0057	10.5			0.0599	207
Patient 1	Serum	715		0.0657	0.0116	5.66			0.0183	204
Patient 1	Serum	736		0.0476	0.0154	3.09			0.0080	202
Patient 1	Serum	764		0.0407	0.0158	2.58			0.0061	200
Patient 1	Serum	793		0.0393	0.0136	2.89			0.0050	197
Patient 1	Serum	827		0.0577	0.0162	3.56			0.0055	195
Patient 1	Serum	918		0.0330	0.0149	2.21			0.0047	187
Patient 1	Serum	942		0.0468	0.0335	1.40			0.0029	185
Patient 1	Serum	988		0.0367	0.0254	1.44			0.0026	181
Patient 1	Serum	1030		0.0437	0.0277	1.58			0.0017	178
Patient 1	Serum	1051		0.0569	0.0288	1.98			0.0037	176
Patient 1	Serum	1072		0.0742	0.0273	2.72			0.0043	174
Patient 1	Serum	1121		0.2180	0.0376	5.80			0.0088	170
Patient 1	Serum	1156		0.1000	0.0428	2.34			0.0044	167
Patient 1	Serum	1184		0.0819	0.0385	2.13			0.0030	165
Patient 1	Serum	1233		0.0695	0.0352	1.97			0.0015	161
Patient 1	Serum	1296		0.0927	0.0472	1.96			0.0076	156
Patient 1	Serum	1352		0.0708	0.0436	1.62				Not stored
Patient 1	Serum	1415		0.0919	0.0486	1.89			0.0059	146
Patient 1	Serum	1479		0.1080	0.0758	1.42			0.0077	140
Patient 1	Serum	1541		0.1390	0.0484	2.87			0.0054	135
Patient 1	Serum	1597		0.1890	0.0397	4.76			0.0042	130
Patient 1	Serum	1646		0.3070	0.0518	5.93			0.0047	126
Patient 1	Serum	1709		0.4520	0.0496	9.11			0.0124	121
Patient 1	Serum	1737		0.5150	0.0370	13.9				Not stored
Patient 1	Serum	1779		0.7580	0.0233	32.5			0.0145	115
Patient 1	Serum	1821		1.6900	0.0181	93.4	10.77		0.0341	112
Patient 1	Serum	1843		2.0600	0.0129	160			0.0486	110
Patient 1	Serum	1864		0.4660	0.0096	48.5			0.0105	108
Patient 1	Serum	1891		0.0778	0.0115	6.77		0.76		Not stored
Patient 1	Serum	1926		0.0543	0.0111	4.89				Not stored
Patient 1	Serum	1944		0.0409	0.0129	3.17				Not stored
Patient 1	Serum	1975		0.0455	0.0101	4.50				Not stored
Patient 1	Serum	2010		0.1740	0.0120	14.5			0.0032	96
Patient 1	Serum	2033		0.1210	0.0110	11.0			0.0031	94
Patient 1	Serum	2053		0.0721	0.0120	6.01				Not stored
Patient 1	Serum	2087		0.2220	0.0117	19.0				Not stored
Patient 1	Serum	2108		0.5300	0.0095	55.7				Not stored
Patient 1	Serum	2143		0.4940	0.0080	61.6		0.58		Not stored
Patient 1	Serum	2171		3.5400	0.0090	396			0.0546	83
Patient 1	Serum	2182		3.2600	0.0069	472			0.0578	82
Patient 1	Serum	2213		0.3770	0.0051	73.9				Not stored
Patient 1	Serum	2234		0.0773	0.0063	12.3				Not stored
Patient 1	Serum	2297		0.1480	0.0058	25.50				Not stored
Patient 1	Serum	2321		0.1150	0.0050	23.1				Not stored
Patient 1	Serum	2353		0.1500	0.0046	32.5		0.15		Not stored
Patient 1	Serum	2381		0.1820	0.0028	65.5				Not stored
Patient 1	Serum	2409		0.2510	0.0030	84.2				Not stored
Patient 1	Serum	2437		0.2940	0.0026	114		0.16	0.0066	60
Patient 1	Serum	2460		0.4050	0.0020	200			0.0084	59
Patient 1	Serum	2500		0.4430	0.0031	144			0.0095	55
Patient 1	Serum	2528		0.5010	0.0026	193				Not stored
Patient 1	Serum	2556		0.5670	0.0030	190			0.0109	51
Patient 1	Serum	2581		0.3300	0.0038	85.9			0.0067	48
Patient 1	Serum	2605		0.6020	0.0030	198		0.10	0.0090	46

Table S2: Overview of all collected samples and diagnostic tests performed on each sample. ***(2/6)***

Patient 1	Serum	2640		0.7010	0.0028	250			0.0173	44
Patient 1	Serum	2675		1.2800	0.0027	478			0.0202	41
Patient 1	Serum	2689		1.9100	0.0025	761			0.0274	39
Patient 1	Serum	2717		1.5700	0.0020	805			0.0209	37
Patient 1	Serum	2745		1.4700	0.0020	721			0.0199	35
Patient 1	Serum	2773		1.8400	0.0017	1060			0.0339	32
Patient 1	Serum	2801		2.8700	0.0021	1380			0.0499	30
Patient 1	Serum	2812		3.3800	0.0015	2210			0.0829	29
Patient 1	Serum	2823		2.9800	0.0017	1810	2.43	0.04		Not stored
Patient 1	Serum	2837							0.0788	27
Patient 1	Serum	2852		0.4400	<0.001	-				Not stored
Patient 1	Serum	2892		<0.0012	<0.001	-			0.0025	23
Patient 1	Serum	2915		<0.0012	<0.001	-			0.0021	21
Patient 1	Serum	2948		<0.0012	<0.001	-	12.35		0.0012	18
Patient 1	Serum	2977		<0.0012	<0.001	-			0.0009	15
Patient 1	Serum	3004		<0.0012	<0.001	-			0.0008	13
Patient 1	Serum	3032		<0.0012	<0.001	-	13.48	<0.04	0.0008	11
Patient 1	Serum	3060		0.0012	<0.001	-		<0.04	0.0010	9
Patient 1	Serum	3088		0.0015	<0.001	-	12.14	<0.04	0.0013	6
Patient 1	Serum	3113		0.0016	<0.001	-			0.0009	4
Patient 1	Serum	3144		0.0013	<0.001	-			0.0010	2
Patient 1	Serum	3172		0.0021	<0.001	-			0.0014	1
Patient 1	Serum	3207		0.0034	<0.001	-	9.72	<0.04	0.0021	1
Patient 1	Serum	3249		0.0042	<0.001	-			0.0024	1
Patient 1	Serum	3305		0.0275	<0.001	-			0.0028	1
Patient 2	Serum	0	34.3	0.0551	0.0018	30.6				Not stored
Patient 2	Serum	81	Traces	<0.0012	<0.001	-				Not stored
Patient 2	Serum	139		0.0025	<0.001	-			0.8176	287
Patient 2	Serum	216	Traces	0.0014	0.0015	0.93			0.7857	281
Patient 2	Serum	255	Traces	0.0038	0.0026	1.46				Not stored
Patient 2	Serum	292	Traces	0.0024	0.0013	1.85			0.5623	274
Patient 2	Serum	327	Traces	0.0027	0.0017	1.59			0.3214	271
Patient 2	Serum	355	Traces	0.0037	0.0039	0.95			0.1843	269
Patient 2	Serum	411	Undetectable	0.0082	0.0067	1.22				Not stored
Patient 2	Serum	460	Undetectable	0.0051	0.0031	1.65				Not stored
Patient 2	Serum	516	Traces	0.0053	0.0054	0.98				Not stored
Patient 2	Serum	581	Undetectable	0.0042	0.0050	0.84				Not stored
Patient 2	Serum	635		0.0040	0.0044	0.91				Not stored
Patient 2	Serum	702	Undetectable	0.0045	0.0049	0.92				Not stored
Patient 2	Serum	754	Undetectable	0.0073	0.0065	1.12				Not stored
Patient 2	Serum	824	Undetectable	0.0064	0.0065	0.98			0.1150	230
Patient 2	Serum	887	Undetectable	0.0187	0.0102	1.83			0.1991	225
Patient 2	Serum	945	Undetectable	0.0193	0.0104	1.86			0.1880	220
Patient 2	Serum	999		0.0185	0.0110	1.68			0.3261	215
Patient 2	Serum	1062	Undetectable	0.0173	0.0099	1.75			0.1561	210
Patient 2	Serum	1116	Undetectable	0.0253	0.0153	1.65			0.1096	206
Patient 2	Serum	1161	Undetectable	0.0182	0.0110	1.65			0.2984	202
Patient 2	Serum	1216	Undetectable	0.0193	0.0098	1.97			0.1036	197
Patient 2	Serum	1328	Undetectable	0.0243	0.0112	2.17			0.3221	188
Patient 2	Serum	1347	Undetectable	0.0131	0.0069	1.90			0.4118	186
Patient 2	Serum	1384	Undetectable							Not stored
Patient 2	Serum	1405	Undetectable							Not stored
Patient 2	Serum	1434	Undetectable	0.0075	0.0081	0.93	5.12	0.98	0.0108	179
Patient 2	Serum	1446		0.0072	0.0054	1.33			0.0127	178
Patient 2	Serum	1459	Undetectable				6.31	1.05		Not stored
Patient 2	Serum	1511	Undetectable							Not stored
Patient 2	Serum	1525	Undetectable							Not stored
Patient 2	Serum	1552	Undetectable	0.0102	0.0023	4.43			0.0138	169
Patient 2	Serum	1616	Traces	0.0118	0.0031	3.81			0.0812	164
Patient 2	Serum	1692	Traces	0.0112	0.0035	3.20			0.0992	158
Patient 2	Serum	1790	Traces	0.0101	0.0042	2.40		0.84		Not stored
Patient 2	Serum	1867	Traces	0.0103	0.0044	2.34	9.76			Not stored
Patient 2	Serum	1922	Traces	0.0117	0.0034	3.44				Not stored
Patient 2	Serum	2000	Traces	0.0135	0.0047	2.87				Not stored
Patient 2	Serum	2091	Traces	0.0105	0.0032	3.28		1.06		Not stored
Patient 2	Serum	2182	Traces					1.28		Not stored
Patient 2	Serum	2268	Traces	0.0125	0.0055	2.27				Not stored

Table S2: Overview of all collected samples and diagnostic tests performed on each sample. *(3/6)*

Patient 2	Serum	2352	Traces	0.0188	0.0040	4.70		2		Not stored
Patient 2	Serum	2427	Traces	0.0352	0.0037	9.59		3.8		Not stored
Patient 2	Serum	2496	8	0.0401	0.0029	13.70				Not stored
Patient 2	Serum	2510	2.7	0.0215	0.0011	19.00		2.86		Not stored
Patient 2	Serum	2517	2.2	0.0209	0.0011	18.70				Not stored
Patient 2	Serum	2538	Traces	0.0157	0.0013	12.50				Not stored
Patient 2	Serum	2575	Traces	0.0134	0.0013	10.50				Not stored
Patient 2	Serum	2596	Traces	0.0129	0.0011	11.30				Not stored
Patient 2	Serum	2616	Traces	0.0120	0.0011	11.40				Not stored
Patient 2	Serum	2637	Traces	0.0126	0.0012	10.30				Not stored
Patient 2	Serum	2665	Traces	0.0143	0.0012	11.70				Not stored
Patient 2	Serum	2693	Traces	0.0158	0.0012	13.60		1.95		Not stored
Patient 2	Serum	2721	Traces	0.0145	0.0014	10.20		2.41		Not stored
Patient 2	Serum	2749	Traces	0.0130	<0.001	-		2.41		Not stored
Patient 2	Serum	2777	Traces	0.0137	0.0011	12.00		2.5		Not stored
Patient 2	Serum	2805	Traces	0.0143	0.0016	8.94		2.99		Not stored
Patient 2	Serum	2833	Traces	0.0245	0.0017	14.10		3.59	3.2165	63
Patient 2	Serum	2861	3.4	0.0206	0.0013	16.50		3.58	3.0279	60
Patient 2	Serum	2889	3.9	0.0225	0.0013	17.20		3.87		Not stored
Patient 2	Serum	2917	Traces	0.0117	0.0019	6.22		1.5	0.9618	56
Patient 2	Serum	2945	Traces	0.0109	0.0027	4.01		1.3	0.8992	53
Patient 2	Serum	2972	Traces	0.0013	0.0011	1.23		0.95	0.5428	51
Patient 2	Serum	2994	Traces	0.0028	0.0021	1.34		0.63		Not stored
Patient 2	Serum	3029	Traces	0.0028	0.0022	1.29		0.64	0.0883	46
Patient 2	Serum	3056	Traces	0.0019	0.0013	1.45		0.71	1.2330	44
Patient 2	Serum	3077	Traces	0.0025	0.0014	1.84		1.01	0.9370	42
Patient 2	Serum	3105	Traces	0.0045	0.0022	2.08		1.67	0.5428	40
Patient 2	Serum	3140	3	0.0050	0.0023	2.16		2.84	2.8126	37
Patient 2	Serum	3177	5.2	0.0033	0.0017	1.93		4.83	3.8150	34
Patient 2	Serum	3196	8.7	0.0028	0.0023	1.23		9.1	5.5678	32
Patient 2	Serum	3224	10.8	0.0022	0.0027	0.82		10.31	6.4414	30
Patient 2	Serum	3253	13.1	0.0017	0.0029	0.59	3.23	11.98	14.4914	28
Patient 2	Serum	3274	16.8	<0.0012	0.0021	-		15.51	17.4094	26
Patient 2	Serum	3296					2.83	15.06		Not stored
Patient 2	Serum	3308	19.5					16.39	19.5000	23
Patient 2	Serum	3315	19.5					16.33	24.4473	22
Patient 2	Serum	3329					2.25	17.16		Not stored
Patient 2	Serum	3343	13.6					12.26		Not stored
Patient 2	Serum	3357	3.7					3.12	3.7492	19
Patient 2	Serum	3371	Traces					0.97	3.5912	18
Patient 2	Serum	3399	Traces	<0.0012	<0.001	-	5.07	0.09	0.1399	15
Patient 2	Serum	3427	Undetectable	<0.0012	<0.001	-	7.2	<0.04	0.0327	13
Patient 2	Serum	3455	Undetectable	<0.0012	<0.001	-	8.61		0.0247	11
Patient 2	Serum	3483	Undetectable	<0.0012	<0.001	-		<0.04	0.0010	8
Patient 2	Serum	3511		<0.0012	<0.001	-	9.25		0.0010	6
Patient 2	Serum	3539		<0.0012	<0.001	-		<0.04	Undetectable	4
Patient 2	Serum	3563		<0.0012	<0.001	-			Undetectable	2
Patient 2	Serum	3589	Undetectable	<0.0012	<0.001	-	9.94	<0.04	Undetectable	1
Patient 2	Serum	3623	Undetectable	<0.0012	<0.001	-			Undetectable	1
Patient 2	Serum	3651	Undetectable	<0.0011	<0.001	-			Undetectable	1
Patient 2	Serum	3679		<0.0011	<0.001	-			Undetectable	1
Patient 2	Serum	3703		<0.0011	<0.001	-	9.77		Undetectable	1
Patient 3	Serum	0	Traces							Not stored
Patient 3	Serum	288		0.2450	0.0010	245			0.0855	311
Patient 3	Serum	377		0.0190	0.0018	10.6			0.0201	303
Patient 3	Serum	1087		0.5650	0.0040	141				Not stored
Patient 3	Serum	1121		0.0029	0.0016	1.81			0.0117	241
Patient 3	Serum	1142		0.0071	0.0018	3.94			0.0348	239
Patient 3	Serum	1170		<0.0012	0.0015	-			0.0015	237
Patient 3	Serum	1199		<0.0013	<0.001	-			0.0085	235
Patient 3	Serum	1225		<0.0013	<0.001	-			0.0008	232
Patient 3	Serum	1253		<0.0013	0.0013	-			0.0195	230
Patient 3	Serum	1280		<0.0013	0.0013	-			0.0254	228
Patient 3	Serum	1282		<0.0013	0.0014	-			0.0266	228
Patient 3	Serum	1308		<0.0013	0.0011	-			0.0045	226
Patient 3	Serum	1332		<0.0012	<0.0011	-			0.0242	224
Patient 3	Serum	1395		<0.0012	<0.0011	-			0.0137	218

Table S2: Overview of all collected samples and diagnostic tests performed on each sample. ***(4/6)***

Patient 3	Serum	1450		<0.0012	<0.0011	-			0.0176	214
Patient 3	Serum	1506		<0.0012	0.0012	-			0.0112	209
Patient 3	Serum	1562		0.002	0.0021	-			0.0049	204
Patient 3	Serum	1618		<0.0012	0.0011	-				Not stored
Patient 3	Serum	1674		<0.0012	0.0017	-			0.0120	195
Patient 3	Serum	1728		<0.0013	0.0015	-			0.0064	191
Patient 3	Serum	1842		<0.0013	0.001	-			0.0082	181
Patient 3	Serum	1898		<0.0011	0.0013	-			0.0100	176
Patient 3	Serum	1954		<0.0011	0.0011	-			0.0070	172
Patient 3	Serum	2010		0.0016	0.0011	1.45			0.0161	167
Patient 3	Serum	2066		<0.0012	0.0015	-			0.0034	162
Patient 3	Serum	2122		0.0015	0.0017	0.88				Not stored
Patient 3	Serum	2172		0.0017	0.0014	1.21			0.0148	154
Patient 3	Serum	2235		0.0024	0.0019	1.26				Not stored
Patient 3	Serum	2291		0.0048	0.0013	3.69				Not stored
Patient 3	Serum	2354		0.0068	0.0011	6.18				Not stored
Patient 3	Serum	2410		0.0108	<0.900	-				Not stored
Patient 3	Serum	2438		0.0387	0.0011	35.2				Not stored
Patient 3	Serum	2472		0.0627	0.0011	57.0				Not stored
Patient 3	Serum	2522		0.0642	0.0012	53.5				Not stored
Patient 3	Serum	2529		0.0935	0.0011	85.0				Not stored
Patient 3	Serum	2592		0.0974	<0.0009	-				Not stored
Patient 3	Serum	2599		0.0979	<0.0009	-				Not stored
Patient 3	Serum	2613		0.0878	<0.0009	-				Not stored
Patient 3	Serum	2620		0.0880	<0.0009	-				Not stored
Patient 3	Serum	2641		0.1180	<0.0009	-				Not stored
Patient 3	Serum	2647		0.1210	<0.0009	-				Not stored
Patient 3	Serum	2662		0.1460	<0.0009	-				Not stored
Patient 3	Serum	2680		0.1060	<0.0009	-				Not stored
Patient 3	Serum	2696		0.1110	<0.0009	-				Not stored
Patient 3	Serum	2723		0.0966	<0.0009	-	1.76			Not stored
Patient 3	Serum	2738		0.0012	<0.0009	-				Not stored
Patient 3	Serum	2759		<0.0011	<0.0009	-				Not stored
Patient 3	Serum	2780		<0.0011	<0.0009	-				Not stored
Patient 3	Serum	2808		<0.001	<0.001	-				Not stored
Patient 3	Serum	2850		<0.0012	<0.001	-				Not stored
Patient 3	Serum	2865		<0.0012	<0.0009	-				Not stored
Patient 3	Serum	2892		<0.0012	0.0011	-				Not stored
Patient 3	Serum	2920		<0.0012	<0.001	-				Not stored
Patient 3	Serum	2948		<0.0012	<0.001	-				Not stored
Patient 3	Serum	2976		<0.0012	<0.001	-				Not stored
Patient 3	Serum	3004		<0.0012	<0.001	-		<0.04		Not stored
Patient 3	Serum	3032		<0.0012	<0.001	-				Not stored
Patient 3	Serum	3060		<0.0012	<0.001	-				Not stored
Patient 3	Serum	3088		<0.0012	<0.001	-				Not stored
Patient 3	Serum	3116		0.0016	0.0011	1.52				Not stored
Patient 3	Serum	3144		0.0013	0.0011	1.15				Not stored
Patient 3	Serum	3172		0.0014	0.0012	1.22				Not stored
Patient 3	Serum	3201		0.0013	<0.001	-				Not stored
Patient 3	Serum	3228		0.0015	0.0013	1.15				Not stored
Patient 3	Serum	3256		0.0012	<0.001	-				Not stored
Patient 3	Serum	3284		0.0016	0.0017	0.97			0.0140	61
Patient 3	Serum	3312		0.0017	0.0015	1.08				Not stored
Patient 3	Serum	3340		0.0023	0.0022	1.04			0.0148	56
Patient 3	Serum	3368		0.0025	0.0020	1.22			0.0090	54
Patient 3	Serum	3396		0.0029	0.0023	1.26			0.0036	52
Patient 3	Serum	3424		0.0090	0.0055	1.62			0.0066	49
Patient 3	Serum	3451		0.0105	0.0050	2.12			0.0107	47
Patient 3	Serum	3478		0.0115	0.0023	5.11			0.0162	45
Patient 3	Serum	3507		0.0213	0.0030	7.10			0.0301	42
Patient 3	Serum	3535		0.0557	0.0029	19.2			0.0556	40
Patient 3	Serum	3563		0.1620	0.0023	70.4			0.2859	38
Patient 3	Serum	3591		0.3830	0.0029	132	3.08		0.2797	35
Patient 3	Serum	3606		0.6050	0.0027	221			0.5132	34
Patient 3	Serum	3620		1.1700	0.0016	713			0.4357	33
Patient 3	Serum	3640		0.8860	0.0021	432			0.3757	31
Patient 3	Serum	3647		1.4800	0.0017	881.00			0.7554	31

Table S2: Overview of all collected samples and diagnostic tests performed on each sample. *(5/6)*

Patient 3	Serum	3677		2.1400	0.0015	1480.00				Not stored
Patient 3	Serum	3703		2.0700	0.0012	1740				Not stored
Patient 3	Serum	3731		2.0500	<0.001	-				Not stored
Patient 3	Serum	3753		1.7100	<0.001	-			1.1352	22
Patient 3	Serum	3767		1.3600	<0.001	-			0.7150	21
Patient 3	Serum	3781		1.6500	<0.001	-			0.5587	19
Patient 3	Serum	3795		1.2800	<0.001	-	1.05		0.8635	18
Patient 3	Serum	3823		2.3100	<0.001	-	0.79			Not stored
Patient 3	Serum	3845		2.7300	<0.001	-	4.02		0.7117	14
Patient 4	Serum	0	4.1	0.00275	0.00326	0.84				Not stored
Patient 4	Serum	77	1.6							Not stored
Patient 4	Serum	85	1.7	0.00248	0.00332	0.75				Not stored
Patient 4	Serum	126	Traces	0.00259	0.00466	0.56				Not stored
Patient 4	Serum	144					5.9	<0.27		Not stored
Patient 4	Serum	172					5.9	0.32		Not stored
Patient 4	Serum	287					8.8	0.51		Not stored
Patient 4	Serum	321					10.1	0.51		Not stored
Patient 4	Serum	393					9.6	0.6		Not stored
Patient 4	Serum	424					10.6	0.63		Not stored
Patient 4	CSF	511								Not stored
Patient 4	Serum	522	13.8	0.55	0.00325	169.23	22.1	0.44		Not stored
Patient 4	CSF	522								Not stored
Patient 4	CSF	526								Not stored
Patient 4	CSF	530								Not stored
Patient 4	CSF	537								Not stored
Patient 4	CSF	544								Not stored
Patient 4	Serum	550	11.8	0.372	0.00152	244.74	17	0.12		Not stored
Patient 4	CSF	550								Not stored
Patient 4	CSF	557								Not stored
Patient 4	CSF	565								Not stored
Patient 4	Serum	578	17.2	0.439	0.00164	267.68				Not stored
Patient 4	CSF	578								Not stored
Patient 4	Serum	590	21.7							Not stored
Patient 4	Serum	601	19.3	0.331	0.00183	180.87				Not stored
Patient 4	CSF	608								Not stored
Patient 4	Serum	622	5.1	0.0217	0.00139	15.61				Not stored
Patient 4	CSF	636								Not stored
Patient 4	Serum	650	2.3	0.00894	0.00223	4.01				Not stored
Patient 4	CSF	657								Not stored
Patient 4	Serum	678	1.5	0.0091	0.0022	4.14				Not stored
Patient 4	CSF	678								Not stored
Patient 4	Serum	706	1.1	0.0167	0.0031	5.39				Not stored
Patient 4	CSF	706								Not stored
Patient 4	Serum	733	Traces	0.0241	0.0023	10.48	4.04			Not stored
Patient 4	CSF	733								Not stored
Patient 4	Serum	762	1.3	0.051	0.0038	13.42				Not stored
Patient 4	CSF	762								Not stored
Patient 4	CSF	790							0.0077	24
Patient 4	Serum	790	2.8	0.13	0.0024	54.17				Not stored
Patient 4	Serum	812	4.8	0.2530					6.5769	22
Patient 4	Serum	812	4.8	0.253	0.0013	194.62				Not stored
Patient 4	Serum	818							9.7072	22
Patient 4	Serum	819							8.2821	22
Patient 4	CSF	825							0.0207	21
Patient 4	Serum	831	8.9	0.4170					9.9969	21
Patient 4	Serum	831	8.9	0.417	<0.0005	-	14.5	<0.05		Not stored
Patient 4	Serum	845	6.6	0.0843					8.7204	20
Patient 4	Serum	845	6.6	0.0843	<0.0005	-				Not stored
Patient 4	Serum	858		0.0029					7.3772	18
Patient 4	Serum	858		0.0029	<0.0005	-				Not stored
Patient 4	Serum	873	4.1	0.0054					5.0782	17
Patient 4	Serum	873	4.1	0.0054	<0.0005	-				Not stored
Patient 4	Serum	908	2.5	0.0540	0.0057	7.90			4.6344	14
Patient 4	CSF	908							0.0071	14
Patient 4	Serum	937	3.7	0.3620	0.0011	329.09	8.53	<0.05	8.9284	12
Patient 4	CSF	937							0.0071	12

Table S2: Overview of all collected samples and diagnostic tests performed on each sample. ***(6/6)***

Patient 4	Serum	954	8.2	0.7460	0.49733	497.33	11.4	<0.05	9.8108	10
Patient 4	Serum	972	19	1.5600	0.0006	2600.00	21.3	<0.05	16.7891	9
Patient 4	CSF	972							0.0130	9
Patient 4	Serum	986	19.2	2.0000	0.0005	4000.00	23.7	<0.05	19.2918	8
Patient 4	Serum	1000	10.5	0.0254	0.001	25.40	14.1	<0.05	11.9501	7
Patient 4	Serum	1009	6.3	0.0085	0.0006	141.67	11.3	<0.05		Not stored
Patient 4	CSF	1009							0.0138	6
Patient 4	Serum	1041	15.5	1.7400	<0.0005	-	20.3	<0.05	13.3407	3

Table S3. Half-life calculated for each t-Ab administered to each patient. Half-life is calculated from the first time point after therapy stop. Slope is expressed in log10(t-Ab(%))/time(y)). M-protein is determined by MS-MRD.
T-Ab: Therapeutic antibody; s-Ab: supportive antibody

	t-Ab/s-Ab	Slope	Half-life (days)	M-protein (g/L) at stop therapy
**Reference**	*IgG half-life (23 days)*	*-5.494*	*23*	-
**Patient 1**	Daratumumab	-2.557	43	0.007
	Isatuximab	-2.916	43	0.034
	Teclistamab	-4.488	24	0.0008
	Tocilizumab	-5.410	20	-
**Patient 2**	Daratumumab	-1.212	91	0.962
	Teclistamab	*Teclistamab treatment not stopped*		
**Patient 3**	Daratumumab	-1.827	60	0.009
	Teclistamab	0.370	38	0.715
	Tocilizumab	-3.873	28	-
**Patient 4**	Talquetamab	-5.441	20	19.292
	Teclistamab	-2.712	32	8.720
	Tocilizumab	-7.201	15	-
